# Entropy comparison of benzothiadiazole-based covalent-organic frameworks

**DOI:** 10.3389/fchem.2025.1704165

**Published:** 2026-01-02

**Authors:** Soniya Kurian, S. Roy, K. B. Gayathri, K. Jyothish

**Affiliations:** Department of Mathematics, School of Advanced Sciences, Vellore Institute of Technology, Vellore, India

**Keywords:** covalent organic framework, molecular graph, benzothiadiazole, topological indices, entropy

## Abstract

Covalent Organic Frameworks (COFs) are popular photocatalysts that utilize solar energy to generate hydrogen peroxide and evolve hydrogen because of their intrinsic porosity, robust framework, and excellent structural regularity. Benzothiadiazole-based donor-acceptor type COFs, PC-NB and PC-NPB, having distinct π -bridges, influence electron transport and photocatalytic efficiency. Using degree-based topological indices and their entropy analysis, this study attempts to theoretically investigate the covalent organic frameworks PC-NB and PC-NPB to evaluate the structural complexity and stability. By offering an organized method for analyzing molecular graph features, edge partition facilitates the computation of topological indices. The calculated topological indices of the COFs are compared in detail and presented graphically. PC-NPB consistently shows higher values across nearly all degree-based topological indices, suggesting that it has a more connected structure. Additionally, lower entropy values in PC-NB indicate a higher degree of topological regularity and symmetry, which are traits frequently associated with enhanced rigidity, crystallinity, and thermodynamic stability.

## 1 Introduction

Covalent organic frameworks (COFs) are novel porous materials created by covalent bonding of light elements such as carbon, hydrogen, nitrogen, sulfur, and oxygen (Xia et al., 2023). They hold great potential for gas adsorption, molecule separation, drug delivery, catalysis, energy storage, and conversion, because of their high crystallinity, wide surface area, superior stability, and ease of functionalization. For photocatalytic hydrogen peroxide production and water splitting, hundreds of COFs have been designed and synthesized (Huang et al., 2025). Donor-Acceptor (D-A) type COFs are a relatively special class of members within the broad family of COF-based photocatalysts because of the electron donor-acceptor units in their skeleton (Wang et al., 2023). D and A units are arranged alternately in D-A type polymers (Wang et al., 2024). Building a D-A COF can be done in two main ways: either by designing a covalent link with a polar orientation or by including donor and acceptor units into the polymeric backbone. According to recent studies, the photocatalytic efficacy of D-A COFs produced is significantly impacted by both linker composition and linkage orientation. D-C ≡ N-A and D-N ≡ C-A are two types of imine linkage orientations found in D-A-type COFs. The D-C ≡ N-A COFs exhibit better photocatalytic activity compared to the D-N ≡ C-A COFs. Using chromophores such as benzothiadiazole and pyrene as building blocks for COFs enhances the band-gap structure and light absorption (Li et al., 2021; Nagar et al., 2025).

For the study, we consider two benzothiadiazole-based covalent organic frameworks. PC-NB and PC-NPB are two benzothiadiazole-based covalent organic frameworks synthesized via a Schiff base condensation reaction, with benzothiadiazole serving as the acceptor and pyrene as the donor. Since the imine linkage (C ≡ N) is polar, connecting the carbon atom of the imine linkage to the donor pyrene unit allows better electron delocalization. Thus, this orientation of the imine bond helps in the good photocatalytic performance of PC-NB and PC-NPB. If the connection is flipped, then performance will drop. The benzene ring in the π -bridge between pyrene and benzothiadiazole is what distinguishes PC-NB from PC-NPB. In PC-NB, benzothiadiazole was directly linked to imine linkages, in contrast to PC-NPB, which has additional benzene on the π -bridge. This enhances electron mobility in PC-NPB, which supports the hydrogen ( ${\text{H}}_{2}$ ) production process but hinders the production of hydrogen peroxide. The hydrogen evolution rate was 4.4 times higher in PC-NPB with the diphenyl benzothiadiazole building block ( $15.7\;mmol\;h^{-1}\;g^{-1}$ ) than in PC-NB with non-phenyl-substituted benzothiadiazole. With a hydrogen peroxide generation rate of $1376\;\mu mol\;h^{-1}\;g^{-1}$ , PC-NB outperformed PC-NPB by a factor of 2.8 (Waller et al., 2015; Wang et al., 2020; Zhao et al., 2025).

Despite increasing interest in D-A type COFs, a comprehensive investigation comparing the topological indices and entropy of PC-NB and PC-NPB structures has yet to be conducted, which could elucidate structure-property correlations relevant to their photocatalytic performance. The topological index of a molecular structure is a numerical value that shows the branching pattern and molecular structure in a non-empirical manner. It is related to chemical composition, which demonstrates how chemical structures correlate with a variety of physical, chemical, and biological aspects (Manzoor et al., 2020). Due to the interpretability and simplicity of the calculation, degree-based indices were popular among various topological descriptors. Degree-based indices are useful for differentiating structurally similar molecules and provide insightful information about the underlying molecular complexity. Besides these descriptors, the concept of topological entropy has attracted a great deal of interest as a quantitative indicator of the structural uncertainty of molecular graphs. Entropy calculation of molecular graphs is one of the major approaches to measuring the complexity of rational structures. Such metrics have their roots in Rashevsky, who first proposed the idea of topological information content (Manzoor et al., 2020; Rashevsky, 1961). Combining entropy-based research with topological indices can yield more complex knowledge of molecular topology and how it affects compound behavior.

In this paper, the molecular structures of PC-NB and PC-NPB are translated into molecular graphs, where the atoms are treated as vertices and the bonds between them as edges. A number of degree-based topological indices are then calculated to quantitatively capture key molecular features, including branching patterns, connectivity, and overall structural complexity (Kuzmin and Konovorotskii, 1985). In addition, the corresponding topological entropy values are evaluated, which function as quantitative measures of the inherent uncertainty within the molecular structure. The internal structural organization of PC-NB and PC-NPB can be measured quantitatively and comparably using entropy and topological indices. Furthermore, this work shows how chemical graph theory can be utilized to predict and explain molecular properties performance, in addition to being a tool for defining molecular systems (Augustine and Roy, 2022).

## 2 Materials and methods

In this study, we have considered only simple, connected, and undirected graphs represented by G=(V(G),E(G)) , where V(G) is the vertex set and E(G) is the edge set of graph G . The degree of vertex v , denoted by $d_{v}$ , is the number of edges connected to v . Degree-based topological indices are numerical invariants of molecular graphs that are calculated on the basis of the degree of vertex (Jyothish and Santiago, 2025). Some degree-based topological indices are given in Table 1.

**TABLE 1:** Degree-based topological indices.

Index	Formula
Randić	$R\left(G\right)=\sum_{uv\in E\left(G\right)}\frac{1}{\sqrt{d_{u}d_{v}}}$
Reciprocal randić	$RR\left(G\right)=\sum_{uv\in E\left(G\right)}\sqrt{d_{u}d_{v}}$
Reduced reciprocal randić	$RRR\left(G\right)=\sum_{uv\in E\left(G\right)}\sqrt{\left(d_{u}-1\right)\left(d_{v}-1\right)}$
First zagreb	$M_{1}\left(G\right)=\sum_{uv\in E\left(G\right)}\left(d_{u}+d_{v}\right)$
Second zagreb	$M_{2}\left(G\right)=\sum_{uv\in E\left(G\right)}d_{u}d_{v}$
Reduced second zagreb	$RM_{2}\left(G\right)=\sum_{uv\in E\left(G\right)}\left(d_{u}-1\right)\left(d_{v}-1\right)$
Hyper zagreb	$HM\left(G\right)=\sum_{uv\in E\left(G\right)}{\left(d_{u}+d_{v}\right)}^{2}$
Augmented zagreb	$AZ\left(G\right)=\sum_{uv\in E\left(G\right)}{\left(\frac{d_{u}d_{v}}{d_{u}+d_{v}-2}\right)}^{3}$
Harmonic	$H\left(G\right)=\sum_{uv\in E\left(G\right)}\frac{2}{d_{u}+d_{v}}$
Sum connectivity	$SC\left(G\right)=\sum_{uv\in E\left(G\right)}\frac{1}{\sqrt{d_{u}+d_{v}}}$
Geometric arithmetic	$GA\left(G\right)=\sum_{uv\in E\left(G\right)}\frac{2\sqrt{d_{u}d_{v}}}{d_{u}+d_{v}}$
Inverse sum	$IS\left(G\right)=\sum_{uv\in E\left(G\right)}\frac{d_{u}d_{v}}{d_{u}+d_{v}}$
Albertson	$Alb\left(G\right)=\sum_{uv\in E\left(G\right)}|d_{u}-d_{v}|$
Symmetric division	$AZI\left(G\right)=\sum_{uv\in E\left(G\right)}\frac{d_{u}^{2}+d_{v}^{2}}{d_{u}d_{v}}$
Atom bond connectivity	$ABC\left(G\right)=\sum_{uv\in E\left(G\right)}\sqrt{\frac{d_{u}+d_{v}-2}{d_{u}d_{v}}}$

**FIGURE 1:**
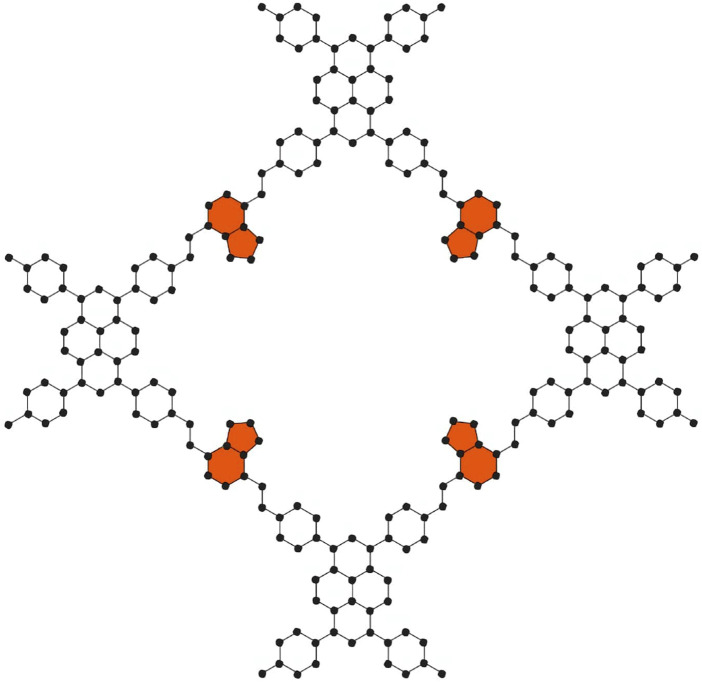
Molecular Structure of PC-NB unit cell.

**FIGURE 2:**
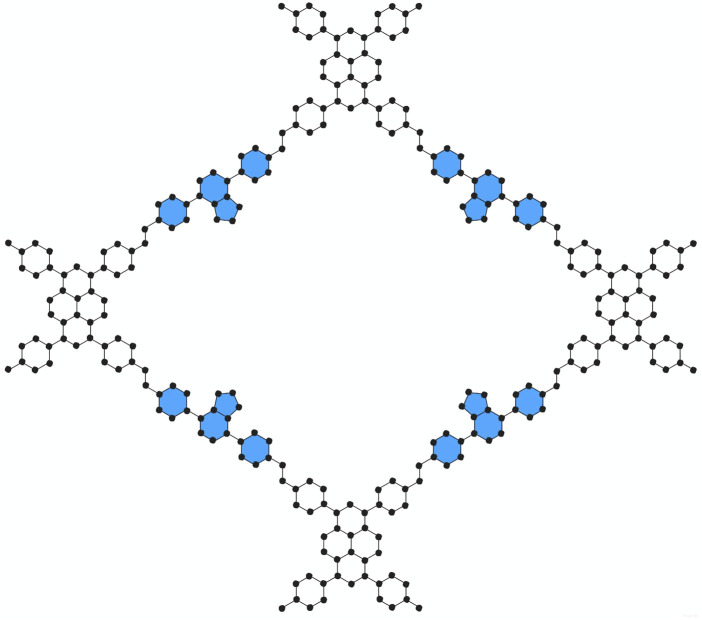
Molecular Structure of PC-NPB unit cell.

Three primary steps comprise our study: (i) building PC-NB and PC-NPB molecular graphs, (ii) using edge partition to calculate degree-based topological indices, and (iii) applying Shannon’s method to calculate entropy values from these indices. A molecular graph is a simple graph that graphically depicts a molecule. Figures 1, 2 depict schematic representations of molecular graphs of the donor-acceptor type covalent organic frameworks PC-NB and PC-NPB, respectively. These graphs provide the foundation for computing degree-based topological indices and entropy metrics. We have used analytical approaches, specifically employing the edge partition methodology for the calculation of topological indices. This partitioning enables a systematic analysis of the graph structure by focusing on different types of connections (Gutman, 2015; Trinajstic, 2018).

Topological indices characterize the topological structure of the molecules to a certain extent. The calculated topological indices of these chemical structures were then thoroughly compared. Entropy is a parameter that measures the structural complexity and stability within the systems. To calculate entropy, we use Shannon’s method by creating the probability function using degree-based topological indices. Entropy measured using the topological index X is given by:

$$E_{X}\left(G\right)=\log\left(X\left(G\right)\right)-\frac{1}{X\left(G\right)}\left(\underset{e\in E\left(G\right)}{\sum }f\left(e\right)\log\left(f\left(e\right)\right)\right)$$ (1)

where E(G) is the set of all edges in the graph G and f(e) is the local value of the index at edge e . Connectivity properties are quantified by degree-based topological indices, and entropy measures derived from these indices offer additional information on the regularity, disorder, or complexity of molecular graphs (Jyothish et al., 2025). Molecular structures were described using KingDraw, while calculations and plotting were done using MATLAB software.

## 3 Results

We consider two benzothiadiazole-based COFs, namely PC-NB, which is directly linked to the imine linkage, and PC-NPB, which has additional benzene in the π -bridge between the imine linkage and the benzothiadiazole unit. The basic unit cells are merged into a rectangular mesh with m rows and n columns to form the 2D COF sheets. In this section, generalized formulations of several degree-based topological indices will be constructed using the edge partition approach. Edge partitions of a molecular graph G are done on the basis of the degrees of the end vertices of each edge of G (Gayathri and Roy, 2025; Govardhan et al., 2024). The PC-NB and PC-NPB sheets with m rows and n columns are denoted by $G_{1}\left(m,n\right)$ and $G_{2}\left(m,n\right)$ , respectively, where m,n≥1 . $G_{1}\left(m,n\right)$ consists of 132nm+44n+44m vertices and 158nm+51n+51m edges, and $G_{2}\left(m,n\right)$ comprises 180nm+44n+44m vertices and 214nm+51n+51m edges. The edge partitions of the COFs are given in Table 2.

**TABLE 2:** Edge-partitions of PC-NB and PC-NPB structures.

Edge-partition	$G_{1}$	$G_{2}$
(1, 3)	4m+4n	4m+4n
(2, 2)	40mn+10m+10n	56mn+10m+10n
(2, 3)	80mn+24m+24n	112mn+24m+24n
(3, 3)	38mn+13m+13n	46mn+13m+13n

### 3.1 Degree-based topological indices of COFs: computation and comparison

This section presents and analyzes the computed degree-based topological indices for the molecular graphs of the covalent organic frameworks PC-NB and PC-NPB. We formulate expressions for each index corresponding to each structure using the edge partitions described in Table 2, which we articulate as Theorems.

Theorem 3.1 Let $G_{1}\left(m,n\right)$ be the molecular graph of the PC-NB structure with dimensions m and n, where m,n≥1 . Then,

1.
$R\left(G_{1}\right)=65.3265mn+21.4407m+21.4407n$

2.
$RR\left(G_{1}\right)=389.9592mn+124.716m+124.716n$

3.
$RRR\left(G_{1}\right)=229.1371n+69.9411m+69.9411n$

4.
$M_{1}\left(G_{1}\right)=788mn+254m+254n$

5.
$M_{2}\left(G_{1}\right)=982mn+313m+313n$

6.
$RM_{2}\left(G_{1}\right)=352mn+110m+110n$

7.
$HM\left(G_{1}\right)=4008mn+1292m+1292n$

8.
$AZ\left(G_{1}\right)=1392.8438mn+433.5781m+433.5781n$

9.
$H\left(G_{1}\right)=64.6667mn+104.6667m+104.6667n$

10.
$SC\left(G_{1}\right)=71.2905mn+23.0404m+23.0404n$

11.
$GA\left(G_{1}\right)=156.3837mn+49.9792m+49.9792n$

12.
$IS\left(G_{1}\right)=193mn+61.3m+61.3n$

13.
$Alb\left(G_{1}\right)=80mn+32m+32n$

14.
$AZI\left(G_{1}\right)=329.3333mn+111.3333m+111.3333n$

15.
$ABC\left(G_{1}\right)=110.1861mn+35.9743m+35.9743n$

Proof. Let $G_{1}\left(m,n\right),m,n\geq 1$ be the molecular graph of the covalent organic framework PC-NB with $n\left(V\left(G_{1}\right)\right)=132mn+44m+44n$ and $n\left(E\left(G_{1}\right)\right)=158mn+51m+51n$ . Then by using the edge partitions given in Table 2 in the definitions of degree-based topological indices in Table 1, we get the following results.

1.
$\begin{array}{cc} R\left(G_{1}\right) & =\left(4m+4n\right)\frac{1}{\sqrt{1\times 3}}+\left(40mn+10m+10n\right)\frac{1}{\sqrt{2\times 2}}+\left(80mn+24m+24n\right)\frac{1}{\sqrt{2\times 3}}\\ & +\left(38mn+13m+13n\right)\frac{1}{\sqrt{3\times 3}}\\ & =65.3265mn+21.4407m+21.4407n \end{array}$

2.
$\begin{array}{cc} RR\left(G_{1}\right) & =\left(4m+4n\right)\sqrt{1\times 3}+\left(40mn+10m+10n\right)\sqrt{2\times 2}+\left(80mn+24n+24m\right)\sqrt{2\times 3}\\ & +\left(38nm+13n+13m\right)\sqrt{3\times 3}\\ & =389.9592mn+124.716m+124.716n \end{array}$

3.
$\begin{array}{cc} RRR\left(G_{1}\right) & =\left(4m+4n\right)\sqrt{\left(1-1\right)\left(3-1\right)}+\left(40mn+10m+10n\right)\sqrt{\left(2-1\right)\left(2-1\right)}\\ & +\left(80mn+24m+24n\right)\sqrt{\left(2-1\right)\left(3-1\right)}+\left(38mn+13m+13n\right)\sqrt{\left(3-1\right)\left(3-1\right)}\\ & =229.1371n+69.9411m+69.9411n \end{array}$

4.
$\begin{array}{cc} M_{1}\left(G_{1}\right) & =\left(4m+4n\right)\left(1+3\right)+\left(40mn+10m+10n\right)\left(2+2\right)+\left(80mn+24m+24n\right)\left(2+3\right)\\ & +\left(38mn+13m+13n\right)\left(3+3\right)\\ & =788mn+254m+254n \end{array}$

5.
$\begin{array}{cc} M_{2}\left(G_{1}\right) & =\left(4m+4n\right)\left(1\times 3\right)+\left(40mn+10m+10n\right)\left(2\times 2\right)+\left(80mn+24m+24n\right)\left(2\times 3\right)\\ & +\left(38mn+13m+13n\right)\left(3\times 3\right)\\ & =982mn+313m+313n \end{array}$

6.
$\begin{array}{cc} RM_{2}\left(G_{1}\right) & =\left(4m+4n\right)\left(1-1\right)\left(3-1\right)+\left(40mn+10m+10n\right)\left(2-1\right)\left(2-1\right)\\ & +\left(80mn+24m+24n\right)\left(2-1\right)\left(3-1\right)+\left(38mn+13m+13n\right)\left(3-1\right)\left(3-1\right)\\ & =352mn+110m+110n \end{array}$

7.
$\begin{array}{cc} HM\left(G_{1}\right) & =\left(4m+4n\right){\left(1+3\right)}^{2}+\left(40mn+10m+10n\right){\left(2+2\right)}^{2}+\left(80mn+24m+24n\right){\left(2+3\right)}^{2}\\ & +\left(38mn+13m+13n\right){\left(3+3\right)}^{2}\\ & =4008mn+1292m+1292n \end{array}$

8.
$\begin{array}{cc} AZ\left(G_{1}\right) & =\left(4m+4n\right){\left(\frac{1\times 3}{1+3-2}\right)}^{3}+\left(40mn+10m+10n\right){\left(\frac{2\times 2}{2+2-2}\right)}^{3}\\ & +\left(80mn+24m+24n\right){\left(\frac{2\times 3}{2+3-2}\right)}^{3}+\left(38mn+13m+13n\right){\left(\frac{3\times 3}{3+3-2}\right)}^{3}\\ & =1392.8438mn+433.5781m+433.5781n \end{array}$

9.
$\begin{array}{cc} H\left(G_{1}\right) & =\left(4m+4n\right)\frac{2}{\left(1+3\right)}+\left(40mn+10m+10n\right)\frac{2}{\left(2+2\right)}+\left(80mn+24m+24n\right)\frac{2}{\left(2+3\right)}\\ & +\left(38mn+13m+13n\right)\frac{2}{\left(3+3\right)}\\ & =64.6667mn+104.6667m+104.6667n \end{array}$

10.
$\begin{array}{cc} SC\left(G_{1}\right) & =\left(4m+4n\right)\frac{1}{\sqrt{1+3}}+\left(40mn+10m+10n\right)\frac{1}{\sqrt{2+2}}+\left(80mn+24m+24n\right)\frac{1}{\sqrt{2+3}}\\ & +\left(38mn+13m+13n\right)\frac{1}{\sqrt{3+3}}\\ & =71.2905mn+23.0404m+23.0404n \end{array}$

11.
$\begin{array}{cc} GA\left(G_{1}\right) & =\left(4m+4n\right)\left(\frac{2\sqrt{1\times 3}}{1+3}\right)+\left(40mn+10m+10n\right)\left(\frac{2\sqrt{2\times 2}}{2+2}\right)\\ & +\left(80mn+24m+24n\right)\left(\frac{2\sqrt{2\times 3}}{2+3}\right)+\left(38mn+13m+13n\right)\left(\frac{2\sqrt{3\times 3}}{3+3}\right)\\ & =156.3837mn+49.9792m+49.9792n \end{array}$

12.
$\begin{array}{cc} IS\left(G_{1}\right) & =\left(4m+4n\right)\left(\frac{1\times 3}{1+3}\right)+\left(40mn+10m+10n\right)\left(\frac{2\times 2}{2+2}\right)+\left(80mn+24m+24n\right)\left(\frac{2\times 3}{2+3}\right)\\ & +\left(38mn+13m+13n\right)\left(\frac{3\times 3}{3+3}\right)\\ & =193mn+61.3m+61.3n \end{array}$

13.
$\begin{array}{cc} Alb\left(G_{1}\right) & =\left(4m+4n\right)|1-3|+\left(40mn+10m+10n\right)|2-2|+\left(80mn+24m+24n\right)|2-3|\\ & +\left(38mn+13m+13n\right)|3-3|\\ & =80mn+32m+32n \end{array}$

14.
$\begin{array}{cc} AZI\left(G_{1}\right) & =\left(4m+4n\right)\left(\frac{1^{2}+3^{2}}{1\times 3}\right)+\left(40mn+10m+10n\right)\left(\frac{2^{2}+2^{2}}{2\times 2}\right)\\ & +\left(80mn+24m+24n\right)\left(\frac{2^{2}+3^{2}}{2\times 3}\right)+\left(38mn+13m+13n\right)\left(\frac{3^{2}+3^{2}}{3\times 3}\right)\\ & =329.3333mn+111.3333m+111.3333n \end{array}$

15.
$\begin{array}{cc} ABC\left(G_{1}\right) & =\left(4m+4n\right)\sqrt{\frac{1+3-2}{1\times 3}}+(40mn+10m+10n)\sqrt{\frac{2+2-2}{2\times 2}}\\ & +\left(80mn+24m+24n\right)\sqrt{\frac{2+3-2}{2\times 3}}+\left(38mn+13m+13n\right)\sqrt{\frac{3+3-2}{3\times 3}}\\ & =110.1861mn+35.9743m+35.9743n \end{array}$

Theorem 3.2 Let $G_{2}\left(m,n\right)$ be the molecular graph of the PC-NPB structure with dimensions m and n, where m,n≥1 . Then,

1.
$R\left(G_{2}\right)=89.0571mn+21.4407m+21.4407n$

2.
$RR\left(G_{2}\right)=524.3429mn+124.716m+124.716n$

3.
$RRR\left(G_{2}\right)=306.392mn+69.9411m+69.9411n$

4.
$M_{1}\left(G_{2}\right)=1060mn+254m+254n$

5.
$M_{2}\left(G_{2}\right)=1310mn+313m+313n$

6.
$RM_{2}\left(G_{2}\right)=464mn+110m+110n$

7.
$HM\left(G_{2}\right)=5352mn+1292m+1292n$

8.
$AZ\left(G_{2}\right)=1867.9688mn+433.5781m+433.5781n$

9.
$H\left(G_{2}\right)=88.1333mn+20.9333m+20.9333n$

10.
$SC\left(G_{2}\right)=96.8673mn+23.0404m+23.0404n$

11.
$GA\left(G_{2}\right)=211.7371mn+49.9792m+49.9792n$

12.
$IS\left(G_{2}\right)=259.4mn+61.3m+61.3n$

13.
$Alb\left(G_{2}\right)=112mn+32m+32n$

14.
$AZI\left(G_{2}\right)=446.6667mn+111.3333m+111.3333n$

15.
$ABC\left(G_{2}\right)=149.4606mn+35.9743m+35.9743n$

Proof. Let $G_{2}\left(m,n\right),m,n\geq 1$ be the molecular graph of the covalent organic framework PC-NPB with $n\left(V\left(G_{2}\right)\right)=180mn+44m+44n$ and $n\left(E\left(G_{2}\right)\right)=214mn+51m+51n$ . Then by using the edge partitions given in Table 2 in the definitions of degree-based topological indices in Table 1, we get the following results.

1.
$\begin{array}{cc} R\left(G_{2}\right) & =\left(4m+4n\right)\frac{1}{\sqrt{1\times 3}}+\left(56mn+10m+10n\right)\frac{1}{\sqrt{2\times 2}}+\left(112mn+24m+24n\right)\frac{1}{\sqrt{2\times 3}}\\ & +\left(46mn+13m+13n\right)\frac{1}{\sqrt{3\times 3}}\\ & =89.0571mn+21.4407m+21.4407n \end{array}$

2.
$\begin{array}{cc} RR\left(G_{2}\right) & =\left(4m+4n\right)\sqrt{1\times 3}+\left(56mn+10m+10n\right)\sqrt{2\times 2}+\left(112mn+24n+24m\right)\sqrt{2\times 3}\\ & +\left(46nm+13n+13m\right)\sqrt{3\times 3}\\ & =524.3429mn+124.716m+124.716n \end{array}$

3.
$\begin{array}{cc} RRR\left(G_{2}\right) & =\left(4m+4n\right)\sqrt{\left(1-1\right)\left(3-1\right)}+\left(56mn+10m+10n\right)\sqrt{\left(2-1\right)\left(2-1\right)}\\ & +\left(112mn+24m+24n\right)\sqrt{\left(2-1\right)\left(3-1\right)}+\left(46mn+13m+13n\right)\sqrt{\left(3-1\right)\left(3-1\right)}\\ & =306.392mn+69.9411m+69.9411n \end{array}$

4.
$\begin{array}{cc} M_{1}\left(G_{2}\right) & =\left(4m+4n\right)\left(1+3\right)+\left(56mn+10m+10n\right)\left(2+2\right)+\left(112mn+24m+24n\right)\left(2+3\right)\\ & +\left(46mn+13m+13n\right)\left(3+3\right)\\ & =1060mn+254m+254n. \end{array}$

5.
$\begin{array}{cc} M_{2}\left(G_{2}\right) & =\left(4m+4n\right)\left(1\times 3\right)+\left(56mn+10m+10n\right)\left(2\times 2\right)+\left(112mn+24m+24n\right)\left(2\times 3\right)\\ & +\left(46mn+13m+13n\right)\left(3\times 3\right)\\ & =1310mn+313m+313n. \end{array}$

6.
$\begin{array}{cc} RM_{2}\left(G_{2}\right) & =\left(4m+4n\right)\left(1-1\right)\left(3-1\right)+\left(56mn+10m+10n\right)\left(2-1\right)\left(2-1\right)\\ & +\left(112mn+24m+24n\right)\left(2-1\right)\left(3-1\right)+\left(46mn+13m+13n\right)\left(3-1\right)\left(3-1\right)\\ & =464mn+110m+110n. \end{array}$

7.
$\begin{array}{cc} HM\left(G_{2}\right) & =\left(4m+4n\right){\left(1+3\right)}^{2}+\left(56mn+10m+10n\right){\left(2+2\right)}^{2}+\left(112mn+24m+24n\right){\left(2+3\right)}^{2}\\ & +\left(46mn+13m+13n\right){\left(3+3\right)}^{2}\\ & =5352mn+1292m+1292n. \end{array}$

8.
$\begin{array}{cc} AZ\left(G_{2}\right) & =\left(4m+4n\right){\left(\frac{1\times 3}{1+3-2}\right)}^{3}+\left(56mn+10m+10n\right){\left(\frac{2\times 2}{2+2-2}\right)}^{3}\\ & +\left(112mn+24m+24n\right){\left(\frac{2\times 3}{2+3-2}\right)}^{3}+\left(46mn+13m+13n\right){\left(\frac{3\times 3}{3+3-2}\right)}^{3}\\ & =1867.9688mn+433.5781m+433.5781n \end{array}$

9.
$\begin{array}{cc} H\left(G_{2}\right) & =\left(4m+4n\right)\frac{2}{\left(1+3\right)}+\left(56mn+10m+10n\right)\frac{2}{(2+2)}+\left(112mn+24m+24n\right)\frac{2}{\left(2+3\right)}\\ & +\left(46mn+13m+13n\right)\frac{2}{\left(3+3\right)}\\ & =88.1333mn+20.9333m+20.9333n \end{array}$

10.
$\begin{array}{cc} SC\left(G_{2}\right) & =\left(4m+4n\right)\frac{1}{\sqrt{1+3}}+\left(56mn+10m+10n\right)\frac{1}{\sqrt{2+2}}+\left(112mn+24m+24n\right)\frac{1}{\sqrt{2+3}}\\ & +\left(46mn+13m+13n\right)\frac{1}{\sqrt{3+3}}\\ & =96.8673mn+23.0404m+23.0404n \end{array}$

11.
$\begin{array}{cc} GA\left(G_{2}\right) & =\left(4m+4n\right)\left(\frac{2\sqrt{1\times 3}}{1+3}\right)+\left(56mn+10m+10n\right)\left(\frac{2\sqrt{2\times 2}}{2+2}\right)\\ & +\left(112mn+24m+24n\right)\left(\frac{2\sqrt{2\times 3}}{2+3}\right)+\left(46mn+13m+13n\right)\left(\frac{2\sqrt{3\times 3}}{3+3}\right)\\ & =211.7371mn+49.9792m+49.9792n \end{array}$

12.
$\begin{array}{cc} IS\left(G_{2}\right) & =\left(4m+4n\right)\left(\frac{1\times 3}{1+3}\right)+\left(56mn+10m+10n\right)\left(\frac{2\times 2}{2+2}\right)+\left(112mn+24m+24n\right)\left(\frac{2\times 3}{2+3}\right)\\ & +\left(46mn+13m+13n\right)\left(\frac{3\times 3}{3+3}\right)\\ & =259.4mn+61.3m+61.3n \end{array}$

13.
$\begin{array}{cc} Alb\left(G_{2}\right) & =\left(4m+4n\right)|1-3|+\left(56mn+10m+10n\right)|2-2|+\left(112mn+24m+24n\right)|2-3|\\ & +\left(46mn+13m+13n\right)|3-3|\\ & =112mn+32m+32n \end{array}$

14.
$\begin{array}{cc} AZI\left(G_{2}\right) & =\left(4m+4n\right)\left(\frac{1^{2}+3^{2}}{1\times 3}\right)+\left(56mn+10m+10n\right)\left(\frac{2^{2}+2^{2}}{2\times 2}\right)\\ & +\left(112mn+24m+24n\right)\left(\frac{2^{2}+3^{2}}{2\times 3}\right)+\left(46mn+13m+13n\right)\left(\frac{2^{3}+3^{2}}{3\times 3}\right)\\ & =446.6667mn+111.3333m+111.3333n \end{array}$

15.
$\begin{array}{cc} ABC\left(G_{2}\right) & =\left(4m+4n\right)\sqrt{\frac{1+3-2}{1\times 3}}+\left(56mn+10m+10n\right)\sqrt{\frac{2+2-2}{2\times 2}}\\ & +\left(112mn+24m+24n\right)\sqrt{\frac{2+3-2}{2\times 3}}+\left(46mn+13m+13n\right)\sqrt{\frac{3+3-2}{3\times 3}}\\ & =149.4606mn+35.9743m+35.9743n \end{array}$

### 3.2 Numerical and graphical analysis of topological indices of COFs

**FIGURE 3:**
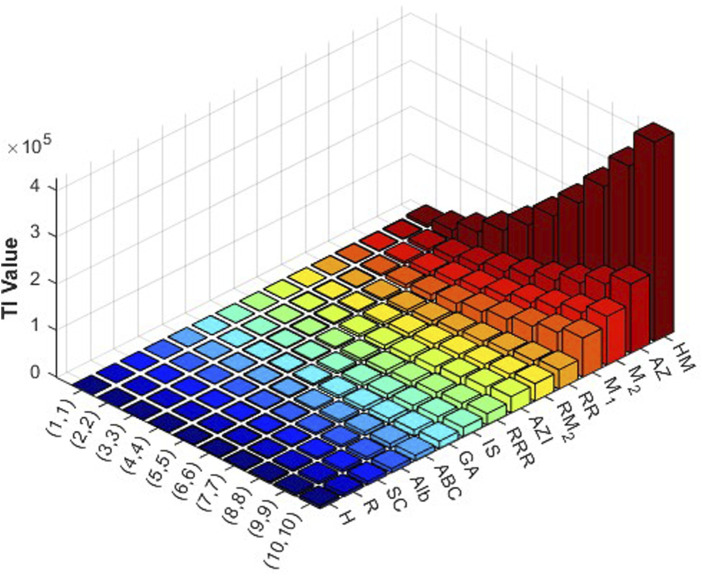
Graphical representation of numerical values of topological indices of PC-NB.

**FIGURE 4:**
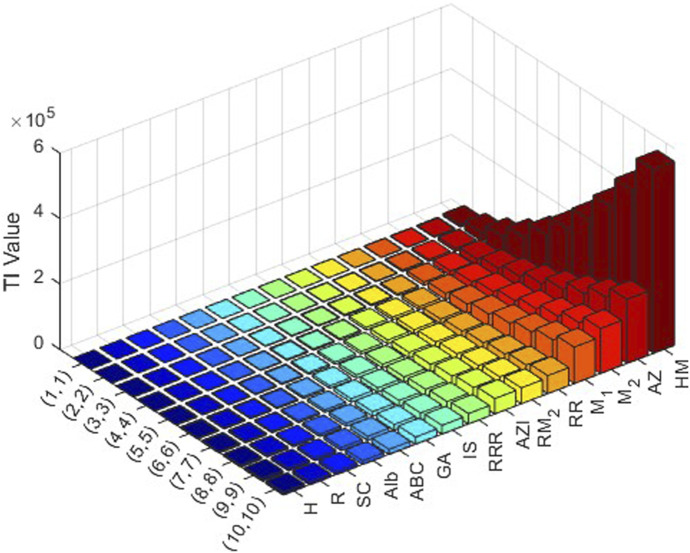
Graphical representation of numerical values of topological indices of PC-NPB.

This section presents the numerical values of the degree-based topological indices for PC-NB and PC-NPB, with variables m and n varying from 1 to 10. Then the obtained data are analyzed comprehensively. The results are summarized in Tables 3, 4 and presented in Figures 3, 4 as three-dimensional bar plots for ease of comparison. The resultant numerical values provide an evaluation of how structural alterations between the two COF structures influence index magnitude and distribution, and these 3D diagrams show the difference between each topological index for a certain structure. The intensity of each value of the topological indices is represented using color mapping.

**TABLE 3:** Numerical values of degree based topological indices of PC-NB.

(m, n)	(1, 1)	(2, 2)	(3, 3)	(4, 4)	(5, 5)	(6,6)	(7,7)	(8,8)	(9,9)	(10,10)
R	108.2079	347.0689	716.5829	1,216.75	1,847.5702	2,609.0434	3,501.1697	4,523.949	5,677.3814	6,961.4669
RR	639.3911	2,058.7005	4,257.9284	7,237.0745	10,996.1391	15,535.1219	20,854.0232	26,952.8428	33,831.5808	41,490.2371
RRR	369.0193	1,196.3128	2,481.8805	4,225.7224	6,427.8384	9,088.2286	12,206.8929	15,783.8315	19,819.0441	24,312.531
${\text{M}}_{1}$	1,296	4,168	8,616	14,640	22,240	31,416	42,168	54,496	68,400	83,880
${\text{M}}_{2}$	1,608	5,180	10,716	18,216	27,680	39,108	52,500	67,856	85,176	104,460
${\text{RM}}_{2}$	572	1,848	3,828	6,512	9,900	13,992	18,788	24,288	30,492	37,400
HM	6592	21,200	43,824	74,464	113,120	159,792	214,480	277,184	347,904	426,640
AZ	2,260	7,305.6875	15,137.0625	25,754.125	39,156.875	55,345.3125	74,319.4375	96,079.25	120,624.75	147,955.9375
H	106.5333	342.4	707.6	1,202.1333	1,826	2,579.2	3,461.7333	4,473.6	5614.8	6,885.3333
SC	117.3712	377.3235	779.8568	1,324.9712	2,012.6666	2,842.9431	3,815.8006	4,931.2391	6,189.2587	7,589.8593
GA	256.3421	825.4515	1707.3283	2901.9724	4409.3838	6229.5626	8362.5088	10,808.2222	13,566.7031	16,637.9512
IS	315.6	1,017.2	2,104.8	3,578.4	5,438	7,683.6	10,315.2	13,332.8	16,736.4	20,526
Alb	144	448	912	1,536	2,320	3,264	4,368	5,632	7,056	8,640
AZI	552	1762.6667	3632	61,660	9346.6667	13,192	17,696	22,858.6667	28,680	35,160
ABC	182.1347	584.6417	1,207.521	2,050.7726	3,114.3965	4,398.3927	5,902.7612	7,627.5019	9,572.615	11,738.1004

**TABLE 4:** Numerical values of degree based topological indices of PC-NPB.

(m, n)	(1, 1)	(2, 2)	(3, 3)	(4, 4)	(5, 5)	(6,6)	(7,7)	(8,8)	(9,9)	(10,10)
R	131.9385	441.9913	930.1584	1,596.4398	2,440.8355	3,463.3454	4,663.9697	6,042.7082	7,599.561	9,334.5281
RR	773.7748	2,596.2352	5,467.3814	9,387.2133	14,355.7309	20,372.9341	27,438.8231	35,553.3978	44,716.6582	54,928.6043
RRR	446.2742	1,505.3322	3,177.174	5,461.7997	8,359.2092	11,869.4026	15,992.3798	20,728.1408	26,076.6857	32,038.0144
${\text{M}}_{1}$	1568	5256	11,064	18,992	29,040	41,208	55,496	71,904	90,432	111,080
${\text{M}}_{2}$	1936	6492	13,668	23,464	35,880	50,916	68,572	88,848	111,744	137,260
${\text{RM}}_{2}$	684	2,296	4,836	8,304	12,700	18,024	24,276	31,456	39,564	48,600
HM	7,936	26,576	55,920	95,968	146,720	208,176	280,336	363,200	456,768	561,040
AZ	2,735.125	9,206.1875	19,413.1875	33,356.125	51,035	72,449.8125	97,600.5625	126,487.25	159,109.875	195,468.4375
H	130	436.2667	918.8	1,577.6	2,412.6667	3,424	4,611.6	5,975.4667	7,515.6	9,232
SC	142.9481	479.6308	1,010.0482	1,734.2003	2,652.0871	3,763.7086	5,069.0648	6,568.1557	8,260.9812	10,147.5415
GA	311.6955	1,046.8654	2,205.5095	3,787.6279	5,793.2205	8,222.2875	11,074.8287	14,350.8442	18,050.334	22,173.2981
IS	382	1,282.8	2,702.4	4,640.8	7,098	10,074	13,568.8	17,582.4	22,114.8	27,166
Alb	176	576	1200	2048	3120	4416	5936	7680	9648	11,840
AZI	669.3333	2,232	4,688	8,037.3333	12,280	17,416	23,445.3333	30,368	38,184	46,893.3333
ABC	221.4092	741.7396	1,560.9912	2,679.164	4,096.258	5,812.2732	7,827.2097	10,141.0673	12,753.8462	15,665.5463

It is evident from examining the Tables 3, 4 that as m and n increase, so do the topological indices. Across almost all indices, PC-NPB consistently exhibits higher values than PC-NB for corresponding (m,n) values. The Randić index for PC-NPB ranges from 131.9385 at (1, 1) to 9334.5281 at (10, 10), while PC-NB has a narrower range from 108.2079 to 6,961.4669. This trend is shared by all topological indices, including HM and H . Among the fifteen degree-based topological indices of covalent organic frameworks (PC-NB and PC-NPB) studied, for every m and n spanning from 1 to 10, the HM index displays the greatest value, while the H index displays the lowest. In 3D plots, Figures 3, 4, the horizontal axis corresponds to (m,n) values and distinct topological indices, and the vertical axis depicts the associated numerical values. The change in color from blue to red signifies an increase in index values.

## 4 Discussion

### 4.1 Degree-based entropy of COFs

In this section, we describe the calculation of entropy measures. The entropies of the corresponding molecular graphs are determined using Equation 1. Here, we have demonstrated the computation of the entropy values of COFs PC-NB and PC-NPB using the First Zagreb Index.

$$\begin{array}{ll} E_{M_{1}}\left(G_{1}\right) & =\log\left(M_{1}\left(G_{1}\right)\right)-\frac{1}{M_{1}\left(G_{1}\right)}\left(\underset{e\in E\left(G_{1}\right)}{\sum }f\left(e\right)\log\left(f\left(e\right)\right)\right)\\ & =\log\left(788mn+254m+254n\right)-\frac{1}{788mn+254m+254n}\left(\left(40mn+10m+10n\right)4\log4\right.+\left(80mn+24m+24n\right)5\log5+\left(38mn+13m+13n\right)6\log6+\left(4m+4n\right)4\log4)=\log\left(788mn+254m+254n\right)-\frac{1}{788mn+254m+254n}\left(\left(160\log4+400\log5+228\log6\right)nm\right.+\left(56\log4+120\log5+78\log6\right)m\left.+\left(56\log4+120\log5+78\log6\right)n\right) \end{array}$$

$$\begin{array}{ll} E_{M_{1}}\left(G_{2}\right) & =\log\left(M_{1}\left(G_{2}\right)\right)-\frac{1}{M_{1}\left(G_{2}\right)}\left(\underset{e\in E\left(G_{2}\right)}{\sum }f\left(e\right)\log\left(f\left(e\right)\right)\right)\\ & =\log\left(1060mn+254m+254n\right)-\frac{1}{1060mn+254m+254n}\left(\left(56mn+10m+10n\right)4\log4\right.+\left(112mn+24m+24n\right)5\log5+\left(46mn+13m+13n\right)6\log6+\left(4m+4n\right)4\log4)=\log\left(1060mn+254m+254n\right)-\frac{1}{1060mn+254m+254n}\left(\left(224\log4+560\log5+276\log6\right)nm\right.\\ & +\left(56\log4+120\log5+78\log6\right)m+\left(56\log4+120\log5+78\log6\right)n) \end{array}$$

Using the procedures described above, the remaining degree-based entropies corresponding to each topological index of PC-NB and PC-NPB are obtained.

### 4.2 Structural complexity and stability of PC-NB and PC-NPB COFs through entropy analysis

**FIGURE 5:**
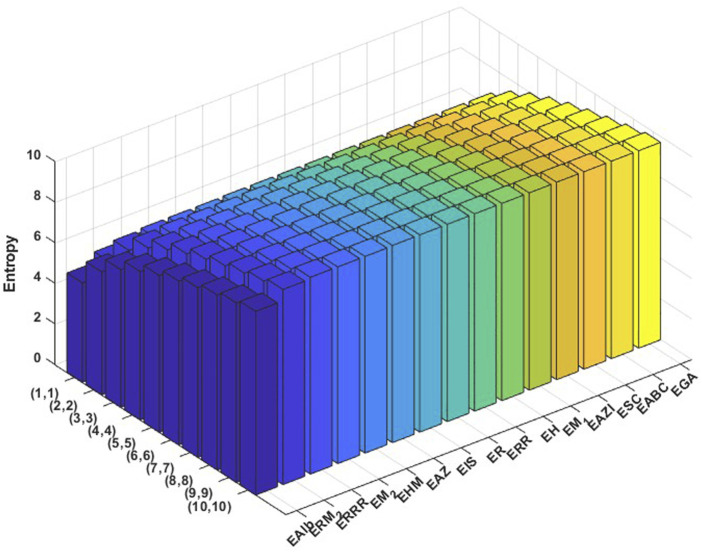
Graphical representation of numerical values of Entropies of PC-NB.

**FIGURE 6:**
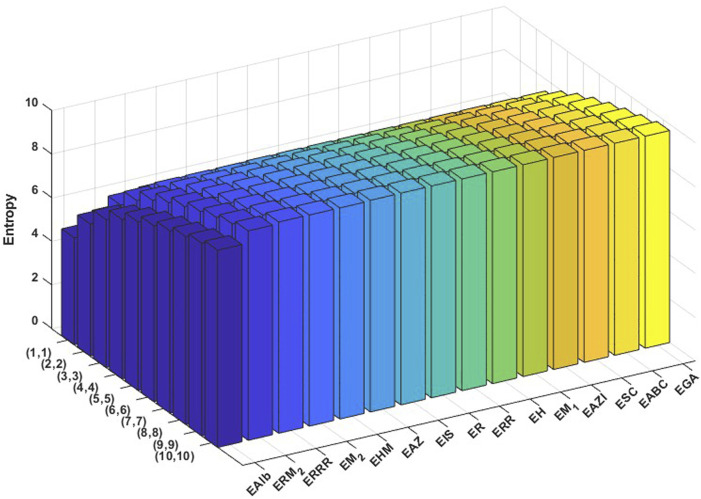
Graphical representation of numerical values of Entropies of PC-NPB.

We conducted a graph-theoretical analysis employing Shannon entropy as a quantitative descriptor to assess the structural complexity and stability of the covalent organic frameworks PC-NB and PC-NPB. Figures 5, 6 show the entropies of PC-NB and PC-NPB graphically. In this context, entropy provides insight into the extent of topological order or disorder within the molecular architecture. Entropy values were assessed for various topological indices across ten different dimensional configurations, indicating successive iterations or depths of structural connectivity (Ezhilan and Varadhan, 2024).

The entropy values for PC-NPB are consistently greater than those for PC-NB across all the entropy values based on different indices and stages of graph structure. In the entropy based on the randić index, PC-NPB shows entropy values between 5.7446 at the (1, 1) iteration and 10.0078 at (10, 10), whereas PC-NB presents a more limited range from 5.5489 to 9.7200. This trend is consistent across all the entropy values based on different indices, including the $M_{1}\left(G\right)$ and $M_{2}\left(G\right)$ . Lower entropy values in PC-NB indicate a higher degree of topological regularity and symmetry, which are characteristics commonly linked to improved thermodynamic stability, rigidity, and crystallinity. This indicates that PC-NB may create more uniform frameworks characterized by well-defined pore channels and predictable stacking interactions. The uniformity of atomic environments in the graph structure suggests enhanced π -electron delocalization, which may lead to improved electronic stability (Mowshowitz and Dehmer, 2012).

The elevated entropy profile of PC-NPB suggests a more irregular and topologically diverse structure, likely due to asymmetric connectivity or a non-uniform distribution of subgraphs. Although these features may compromise overall framework stability, they can also improve functional versatility, such as increased porosity, surface heterogeneity. PC-NPB demonstrates potential for applications that necessitate dynamic host-guest interactions, including molecular sensing, catalysis, and selective adsorption. The entropy analysis indicates a distinct topological difference: PC-NB is characterized by a structurally ordered and potentially more stable framework, whereas PC-NPB displays increased configurational complexity, suggesting improved functional responsiveness. The findings demonstrate the significance of entropy-based metrics in offering an additional viewpoint on structure–property relationships in the rational design of COFs (Shannon, 1948; Vijay et al., 2023).

## 5 Conclusion

This study used topological indices to characterize the covalent organic frameworks, PC-NB and PC-NPB, based on their vertex degrees. Using Shannon’s approach, their entropy values were evaluated for a range of topological indices across ten distinct dimensional configurations. Our calculations reveal that PC-NB is more topologically regular and symmetric than PC-NPB. According to this, PC-NB might provide more homogeneous frameworks with distinct pore channels and consistent stacking interactions, which could result in better electronic stability by enhancing π -electron delocalization. Additionally, the properties of PC-NPB, such as higher porosity and surface heterogeneity, may undermine the overall stability of the framework, but they also offer improved functional adaptability. In the rational design of COFs, the results show the importance of entropy-based metrics in providing an extra perspective on structure–property connections. Hence, the graph-theoretical analysis provides significant insights into the stabilities of the PC-NB and PC-NPB covalent organic frameworks.

## Data Availability

The original contributions presented in the study are included in the article/supplementary material, further inquiries can be directed to the corresponding author.
